# Potential confounders for the effect of high-flow nasal cannula oxygen therapy

**DOI:** 10.1186/s13054-019-2310-3

**Published:** 2019-01-17

**Authors:** Satoshi Yamaga, Shinichiro Ohshimo, Nobuaki Shime

**Affiliations:** Department of Emergency and Critical Care Medicine, Graduate School of Biomedical & Health Sciences, Hiroshima University, 1-2-3 Kasumi, Minami-ku, Hiroshima, 734-8551 Japan

To the Editor:

We read with considerable interest the article published in a recent issue of *Critical Care* by Di Mussi and colleagues [1], who investigated the physiological effects of high-flow nasal canula (HFNC) compared with conventional O_2_ therapy after extubation in patients with chronic obstructive pulmonary disease (COPD) receiving mechanical ventilation. The authors demonstrated that postextubation HFNC significantly decreased neuroventilatory drive and the work of breathing compared with conventional O_2_ therapy. However, several factors that might potentially affect their results should be discussed.

First, although the authors discussed one of the mechanisms related to the decreased work of breathing using HFNC compared with conventional O_2_ therapy, which was associated with the flow-dependent CO_2_ wash-out effect, they did not directly assess the relationship between the variables of work of breathing and the flow rate of HFNC. Various flow rates ranging from 20 to 60 L/min were used in this study, which are inconsistent with previous studies, where the flow rates of HFNC were fixed [2, 3]. A higher flow rate of HFNC potentially increases the CO_2_ wash-out effect and consequently decreases the respiratory effort [4].

Second, the reasons for reintubation in this study were unclear. The authors failed to show a correlation between the work of breathing and the requirement of reintubation; more significant risk factors of reintubation could include other clinical conditions. Shock or disturbed consciousness, masking the effect of HFNC, might be potential confounders in this study.

Finally, we would like to know the differences in clinical characteristics between the patients who benefited from HFNC and those who did not. The authors demonstrated that several patients showed almost no change in neuroventilatory drive and work of breathing independent of the use of HFNC or conventional O_2_ therapy. We speculate that the strength of respiratory drive or the severity of COPD affected these parameters.

In conclusion, we believe that clarification of these issues by the authors would be helpful for a better understanding of the benefit of postextubation HFNC in patients with COPD.

## Abbreviations

COPD: Chronic obstructive pulmonary disease
HFNC: High-flow nasal canula
